# Study protocol for a pragmatic randomised controlled trial in general practice investigating the effectiveness of acupuncture against migraine

**DOI:** 10.1186/1472-6882-8-12

**Published:** 2008-04-14

**Authors:** Jorge Vas, Ángel Rebollo, Emilio Perea-Milla, Camila Méndez, Carlos Ramos Font, Manuel Gómez-Río, Manuel Martín-Ávila, Justo Carbrera-Iboleón, M Dolores Caballero, M Ángeles Olmos, Inmaculada Aguilar, Vicente Faus, Francisco Martos

**Affiliations:** 1 Pain Treatment Unit, Primary Care Center, Dos Hermanas, Spain; 2 Service of Nuclear Medicine, Virgen de las Nieves Hospital, Granada, Spain; 3 Support Research Unit (Network and Cooperative Research Centers of Epidemiology. CIBERESP) Costa del Sol Hospital, Marbella, Spain; 4 Andalusian Public Health System, Sevilla, Spain; 5 Armilla Primary Care Center, Granada, Spain; 6 Valle de Lecrin Primary Care Center, Granada, Spain; 7 Pain Clinic, Virgen de las Nieves Hospital, Granada, Spain; 8 Ugijar Primary Care Center, Granada, Spain; 9 Service of Pharmacology, Costa del Sol Hospital, Marbella, Spain; 10 Department of Pharmacology, Malaga University, Spain

## Abstract

**Background:**

Migraine is a chronic neurologic disease that can severely affect the patient's quality of life. Although in recent years many randomised studies have been carried out to investigate the effectiveness of acupuncture as a treatment for migraine, it remains a controversial issue. Our aim is to determine whether acupuncture, applied under real conditions of clinical practice in the area of primary healthcare, is more effective than conventional treatment.

**Methods/Design:**

The design consists of a pragmatic multi-centre, three-armed randomised controlled trial, complemented with an economic evaluation of the results achieved, comparing the effectiveness of verum acupuncture with sham acupuncture, and with a control group receiving normal care only.

Patients eligible for inclusion will be those presenting in general practice with migraine and for whom their General Practitioner (GP) is considering referral for acupuncture. Sampling will be by consecutive selection, and by randomised allocation to the three branches of the study, in a centralised way following a 1:1:1 distribution (verum acupuncture; sham acupuncture; conventional treatment). Secondly, one patient in three will be randomly selected from each of the acupuncture (verum or sham) groups for a brain perfusion study (by single photon emission tomography). The treatment with verum acupuncture will consist of 8 treatment sessions, once a week, at points selected individually by the acupuncturist. The sham acupuncture group will receive 8 sessions, one per week, with treatment being applied at non-acupuncture points in the dorsal and lumbar regions, using the minimal puncture technique. The control group will be given conventional treatment, as will the other two groups.

**Discussion:**

This trial will contribute to available evidence on acupuncture for the treatment of migraine. The primary endpoint is the difference in the number of days with migraine among the three groups, between the baseline period (the 4 weeks prior to the start of treatment) and the period from weeks 9 to 12. As a secondary aspect, we shall record the index of laterality and the percentage of change in the mean count per pixel in each region of interest measured by the brain perfusion tomography, performed on a subsample of the patients within the real and sham acupuncture groups.

**Trial registration:**

Current Controlled Trials ISRCTN98703707.

## Background

Headache, which is one of the most common causes of patients' seeking attention at neurology clinics, is a highly prevalent health problem and one with considerable socioeconomic impact [1-4]. In Spain, the prevalence of migraine is 16.9% among women and 7.4% among men [5], with 60% of patients having a family background of such headaches [6]. This epidemiologic profile is similar to that reported for other countries [7]. Headache can be considered a social pathology, as it is often discapacitating. A population based study in the USA found that most of the cost provoked by headaches was related to days off work and loss of productivity [8]. It has been estimated that routine activities are limited during 78% of migraine attacks [9]. Spain is believed to lose 13 million working days every year because of migraine [10], and its direct and indirect costs amount to 1076 million Euros, of which 65% is accounted for by indirect costs [5].

There is no curative treatment for migraine, but there do exist various options for pharmacological treatment, and the choice of one type or another depends on the frequency of the attacks, their severity, the patient's activity and tolerance to side effects. In Spain it has been estimated that the most common treatment is applied at the moment of the attack (86.6%); the drugs used when a severe attack occurs include non steroidal anti-inflammatory drugs (NSAIDs) (36.7%), tryptans (28.4%), paracetamol (7.7%) and ergotamine derivatives (7.7%). Patients who suffer repeated attacks are recommended the prophylactic administration of medication such as beta blockers, tricyclic antidepressants and antiepileptic drugs [11]. The effectiveness of these treatments, although it has been corroborated by numerous studies, is limited. For severe cases, it has been estimated that one third of patients fail to respond to the administration of tryptans, which moreover are reserved for those patients who suffer the most severe attacks or who been found to respond poorly to NSAIDs. Prophylactic treatment reduces the frequency of attacks by 50%, but requires a gradual increase of the dose applied and it has an objectifiable benefit of 6 months. Prophylaxis, therefore, reduces but does not eliminate the problem and the need for medication against severe attacks [12]. Tolerance to pharmacological treatment is limited, both for therapy for severe cases and for preventive measures. The main side effects in the former case are gastrointestinal disorders (with NSAIDs) or cardiovascular problems (with ergotamine substances and tryptans). Among the side effects arising from chronic treatment are sedation, fatigue, lack of tolerance to exercise, and weight gain. In addition, there are physiological factors such as pregnancy or old age, and pathological ones such as asthma, ischemic heart disease or hypertension, for which the administration of some of these drugs is contraindicated, and this circumstance also affects the applicability of the therapy in clinical practice [12].

Acupuncture is widely used to treat headache, and can be applied as the sole therapy or as part of a more complex treatment programme [13,14]. Despite its popularity, there persists some controversy as to the differentiation between the specific and the nonspecific effects of acupuncture. A Cochrane review published in 2001 [15] concluded that, in general terms, current evidence supports the value of acupuncture as a treatment for idiopathic headaches, but due to clinical heterogeneity and the low methodological quality of the 26 studies included, it was not possible to make direct recommendations for clinical practice. Fundamentally, some forms of acupuncture seem to be beneficial, but it is unclear as to which treatment strategies (acupuncture points, type of stimulation, frequency, etc.) should be favoured.

The three most recent studies published, during the last two years, report conflicting results. Two studies, which can be classified as belonging to the German strategy of assessing the coverage of acupuncture for certain processes, concluded that both verum acupuncture and sham acupuncture (superficial acupuncture at points not considered to be acupuncture points) are more effective than remaining on the waiting list, untreated [13], but that their results did not differ significantly from those obtained by pharmacological treatment [16]. The third study was a pragmatic one that concluded that acupuncture produces a clinically significant improvement in patients suffering chronic headache, especially in the case of migraine, compared with conventional treatment; moreover, it leads to decreased consumption of medication, visits to the GP and days off work [14]. Endres et al., in a recent review of the subject, concluded that acupuncture treatment for 6 weeks is not inferior to preventive pharmacological treatment for 6 months, but that the use of acupuncture at specific points, needle stimulation and the depth of the puncture are not as important as had previously been thought [17].

The single photon emission computed tomography (SPECT) of brain perfusion is a technique that obtains three-dimensional images of the cerebral distribution of a radiopharmaceutical, reflecting regional brain perfusion [18,19]. SPECT is less expensive than other techniques, is more widely available and is of more general clinical application; moreover, it may enable a semiquantitative analysis of the images [20]. Numerous studies have been made of regional cerebral blood flow (rCBF) in patients with migraine, and hypoperfusion has been found in many cases, with asymmetries in others [21-27]. Indeed, some authors have correlated the abnormalities observed with the severity of the attack [25]. Few studies have made use of brain perfusion SPECT to examine the effects of acupuncture. Lee et al. studied the cerebrovascular response to traditional acupuncture among patients suffering an obstruction of the middle cerebral artery, and compared the results obtained with those produced among 8 healthy volunteer control subjects. Among the patients, there were focal increases in rCBF in the hyperperfused regions that surrounded the ischemic injury, and also in the ipsilateral or contralateral sensory-motor cortex, while the controls presented multiple sites with raised rCBF in each hemisphere, thus showing that among the patients acupuncture produced an activation of neurons that are viable and undergoing cerebral reorganization [28]. Wang and Jia studied the effects of acupuncture on the rCBF of 9 patients with cerebrovascular disease, and on that of 11 healthy subjects, finding significant differences in the cerebral cortex and the contralateral thalamus, in the ipsilateral basal ganglia and in the cerebellum [29]. Newberg et al. investigated changes in rCBF associated with the analgesic effect of acupuncture in 7 patients with chronic pain, and compared these effects with those found in 5 healthy controls. Baseline studies of the thalamus of patients suffering pain presented significant asymmetries, in comparison with the controls. This asymmetry became normalized or inverted after acupuncture. These authors also found significant correlations with changes in the activity of the prefrontal cortex and in the sensory-motor ipsilateral area [30]. Ha-Kawa et al. described the changes in rCBF induced by acupuncture among 3 patients with dystonia, and related these changes to the action of acupuncture on the central nervous system [31].

Acupuncture produces complex changes in the areas of the brain concerned with pain transmission and perception, although the specific details of these effects have yet to be established. The use of neuroimaging techniques enables us to observe changes in cerebral cortical perfusion related to the type of acupuncture applied (verum or sham), and to determine the existence of specific or nonspecific patterns of cortical activation [32].

Summing up, the disparity in results obtained, depending on how the technique is applied, on the diverse therapeutic strategies possible with acupuncture, and on the need to attempt to measure the specific and the nonspecific effects of the technique, led us to design this randomised controlled pragmatic study aimed at investigating the effectiveness of acupuncture applied in primary healthcare centres for patients suffering migraine. Our goals are: a) to assess the effectiveness and safety of acupuncture in the context of standard clinical practice; b) to derive an economic evaluation of the technique; c) to determine the specific and nonspecific effects of the intervention in the study; and d) to examine changes in brain perfusion using SPECT tomography to examine the consequences of the different interventions.

## Methods

### Design

Randomised controlled multicentre pragmatic study, with three arms, to compare the effectiveness of the individualized active acupuncture (Group A), minimal acupuncture (in locations not corresponding to acupuncture points, in different metamers and at a depth of less than 3 mm) (Group B) and conventional treatment (Group C). The patients will be blinded to the two acupuncture treatments. These patients will be assessed and the results analysed by professionals blinded with respect to the allocations of the different treatments. A stratified randomised subsample of groups A and B will be given a SPECT tomography in order to determine possible changes in the cerebral vascular flow provoked by the two different types of acupuncture applied. The duration of the study, per patient, will be 28 weeks, four of which will be prior to randomisation (baseline), followed by 8 weeks of treatment and then 16 weeks' follow up.

### Study period

January 2008-December 2010

### Study subjects

General practitioners at the three primary healthcare clinics taking part in the study, belonging to the public healthcare system in Andalusia (at Armilla, Valle de Lecrín and Ugíjar, all in the province of Granada), will identify potentially eligible patients with migraine symptoms. An independent assessor will interview the participants, perform the screening and instruct the patients in how to complete the headache diary (to be filled in over a period of 4 weeks). The patients will be informed about the procedures of the study and will be requested to sign the informed consent form. They will also be informed about the possible risks associated with the different types of acupuncture (infection, fainting, bruising) and told that that they may end their participation in the study at any time, with no type of penalty or loss of benefits to which they would otherwise be entitled. Although only a subsample will be given the SPECT diagnostic test, all the patients included in the study will be required to sign their informed consent to this test.

The patients included must be at least 18 years old, diagnosed with migraine, in accordance with the principles of the International Headache Society [1] and the recommendations for clinical trials published by the same Society [33], with or without aura, with a frequency of migraine attacks of 2–6 times per month, with a minimum chronicity of one year, the onset of symptoms at an age of less than 50 years, having completed the headache diary, and have signed their informed consent. Criteria for exclusion will include acupuncture during the 12 previous months, incapacity to distinguish between tensional headache and migraine, secondary headaches, contraindication to acupuncture (pregnancy, generalised dermopathy, treatment with anticoagulants, thrombocytopenia) or to the performance of SPECT techniques (cerebrovascular accident, traumatic brain injury, alcohol or drug abuse, severe psychiatric disorders) and inability to complete the questionnaires or to reply to the assessor's questions.

The ethical validity of this study has been assessed and approved by the Andalusian Regional Committee for Clinical Trials, following approval by the Research Committee corresponding to each of the participating centres. The study design complies with the fundamental principles set out in the Helsinki Declaration, as revised in Tokyo (2004), as well as those of the Council of Europe Convention on human rights and biomedicine, and the requirements of Spanish law regarding the field of biomedical research, the protection of personal data, and bioethics. All patients must sign the form of informed consent to clinical research procedures. During the course of the study, audits will be carried out as considered necessary by the corresponding Committee for Research Ethics and by the Quality Control Committee responsible for the centre in question, these being independent of any external audits (by the source of research funding) that may be required.

### Randomisation and treatment allocation

Sampling will be by consecutive selection, in accordance with inclusion-exclusion criteria, over a period of 24 months until the predetermined sample size has been obtained. Randomised allocation to the three arms of the trial will be carried out using an appropriate computer program (EpiDat v 3.1), in a centralised way (by the Research Unit at Hospital Costa del Sol, Malaga), following a 1:1:1 distribution (verum acupuncture: minimal acupuncture: conventional treatment), with a sequence for each clinic in blocks of 9. Secondarily, one patient in three will be randomly selected from each of groups A and B to be given a SPECT brain perfusion study. Neither the centres nor the doctors participating in the trial will take part in the randomisation procedure, and the sequence of the latter will remain concealed until the completion of the trial. The patients who fulfil the criteria for inclusion and who give their informed consent will be included in the trial. After each patient's inclusion, his/her GP will contact the randomisation centre, where the patient will be registered, and the doctor will be told, by phone and by fax, which of the three arms of the trial the patient has been allocated to. This procedure guarantees that the randomisation process will not be influenced by the doctor participating. We shall endeavour to ensure that all participants begin the trial with the same expectations of effectiveness; accordingly, the initial information provided to the patient will state that all the treatments provided in the course of the trial are effective [13,14,16].

### Criteria and procedure for withdrawal of patients from the trial

A patient may be withdrawn from the trial at any time, at his/her own discretion or at that of the researcher. The reasons for interrupting the patient's participation in the trial will be recorded on the summary page of the Data Record Book (DRB). If there is any suspicion of secondary headache or of a malignant pathology, as well as the patient being withdrawn from the trial, he/she will be referred to his/her GP so that a conclusive diagnosis may be made on the basis of relevant complementary tests, or so that referral to a neurologist may be considered.

### Sample size

In calculating the sample size, we assumed a power of 90% and α value of 5%, in order to detect a difference of 1.6 days with migraine with a standard deviation of 2.8 days; this implies a sample size of 72 per group in a design with three equal groups. We propose to recruit 270 patients in order to allow for a 20% withdrawal rate.

### Interventions

The treatment strategies with verum and with sham acupuncture were developed by means of consensus with experienced acupuncture practitioners. All the patients will be allowed to take symptomatic medication to treat headache episodes if necessary, and to continue taking the medication to prevent migraine attacks, following their GPs' recommendations and those of the Headache Study Group of the Spanish Neurological Society [34].

The two groups will receive a total of 8 acupuncture sessions (at a rate of 1 session per week), each lasting 30 minutes, comprising the technique described below and at the locations specified. The acupuncture needles will be sterile, single-use, model S-J2030, made by Seirin Corporation (Shizuoka, Japan) in accordance with EC regulations.

#### A) Verum acupuncture

Individualized treatment on the basis of diagnosis in accordance with traditional Chinese medicine (TCM), which the acupuncture physician may modify in accordance with the evolution of the patient's symptoms. The TCM diagnosis, evolution, points and techniques applied should be recorded in the DRB.

The doctors responsible for the treatment (specialists in acupuncture with a minimum training of 600 hours and a mean experience of 5.7 years) will insert sterile, single-use filiform acupuncture needles, with a length of 10–30 mm and a diameter of 0.20 mm, with the aid of a guide tube at each of the points, after first disinfecting the skin and with the patient in decubitus. The puncture will be made in accordance with the standards of TCM, to a depth of 1–3 cm, depending on the points selected. The insertion will be followed by stimulation performed with bidirectional rotation actions of the needle sheath in order to produce the sensation known as *Deqi*, commonly described as a sensation of irradiation. The needle will be maintained in place for 30 minutes, with 1–3 manipulations per session.

Any adverse reaction or side effect should be recorded in the DRB, with a detailed description, and stating the date of occurrence.

The time dedicated to the patients allocated to each of the groups should be identical. Similarly, the same time should be devoted to pre and post session evaluations.

The patients randomly selected for the SPECT diagnostic test will be given their first session at the Nuclear Medicine Department of the Virgen de las Nieves Hospital. The remaining sessions, as well as those applied to the other patients, will be performed at the healthcare centres participating in the trial.

#### B) Sham acupuncture

The patients who are randomly assigned to this group will be given minimal acupuncture (the real insertion of acupuncture needles, to a depth of less than 3 mm) at 5 bilateral non-acupuncture points located 1.5 cm from the mean dorsal line and lumbar curve, specifically, at the spinal apophysis of the T10, T11, T12, L1 and L3 vertebrae.

The technique will not differ at all from that used for Group A, except that no attempt will be made to produce the *Deqi *sensation.

#### C) Conventional treatment

The patients will be given the conventional treatment prescribed by their GP.

### Brain perfusion tomography (SPECT)

#### Preparation

▪ During the 24 hours prior to the examination, the patient must not consume coffee, colas, energy drinks, alcohol, tobacco or any drugs that might affect the cerebral blood flow.

▪ The procedure will be explained to the patient and his/her capacity to collaborate in the performance of the examination will be assessed.

#### Criteria for exclusion

Patients will be excluded from the trial if they are lactating or have a case history suggesting possible alterations to brain perfusion patterns in the SPECT test (such as cerebrovascular accident, traumatic brain injury, alcohol or drug abuse, or severe psychiatric disorders).

#### Radiopharmaceuticals

The radiopharmaceutical used will be 99mTc-ECD (Neurolite, Bristol-Myers Squibb Pharma Belgium Sprl). The manufacturer's instructions will be followed for reconstitution and labelling of the product. The dose given to each patient will be measured using an activimeter (Capintec CRC-35R, Cleveland Ohio USA) immediately following intravenous administration of the radiopharmaceutical. Its quality will be controlled in accordance with the method described by the manufacturer, to achieve at least the recommended radiochemical purity of 90%.

#### Gammacamera

In carrying out the examination, we will use a triple-head gammacamera (Picker Prism 3000, Cleveland Ohio USA), equipped with collimators for a low-energy and ultra high-resolution neuro-fan beam (Leuhr-NeuroFan), connected to a Picker Odyssey FX image processing workstation.

#### Procedure

▪ Baseline or pre-acupuncture trial:

• With the patient in decubitus, eyes open and ears un-occluded, administer 260 MBq (7 mCi) of 99mTc-ECD intravenously, with the vein canalized at least 10 minutes previously.

• Image acquisition will begin 20 minutes following the injection of the radiopharmaceutical, in accordance with the following parameters: 360° circular orbit, rotation radius of 12.9 cm, step and shoot mode (3°/40 sec/step), 128 × 128 matrix, no zoom, and an energy window of 140 ± 20 KeV. Total acquisition time, 45 minutes.

• After this initial image acquisition, the patients will be given the first acupuncture session (verum or sham), under the following procedure:

1. Group A: verum acupuncture at LI4 and LIV3 (these points were decided upon by consensus among the acupuncture physicians taking part in the trial, and because they are the ones most frequently employed in treating primary headache [35]). The insertion will be followed by stimulation performed with bidirectional rotation actions of the needle sheath in order to produce the *Deqi*. The needle will be maintained in place for 30 minutes, with 1–3 manipulations per session.

2. Group B: sham acupuncture at two bilateral points, located 1.5 cm from the spinal apophysis of the T12 and L1 vertebrae, at a depth of less than 3 mm. The technique will not differ at all from that used for Group A, except that no attempt will be made to produce the *Deqi *sensation.

▪ Post-acupuncture trial:

• 5 minutes before ending the acupuncture session, administer 925 MBq (25 mCi) of 99mTc-ECD intravenously. Image acquisition will begin approximately 30 minutes later, using the same acquisition protocol and with a total acquisition time of 20 minutes.

▪ Post-treatment trial:

• This will be performed at week 12 after randomisation.

• This trial will be carried out with the same dose of radiopharmaceuticals and under acquisition conditions identical to those of the post-acupuncture study.

#### Image processing and analysis

For image reconstruction, will be used a filtered backprojection algorithm, using Low-Pass filter Butterworth type (6.0 and a cutoff frequency of 0.3), and attenuation correction by Chang's method with an attenuation coefficient of 0.11 cm-1. After reconstruction imaging is reoriented in cross sectional planes according orbito-meatal axis (transaxial, saggital and coronal) for study comparison. Imaging will be evaluated both qualitatively and semi-quantitatively. For visual assessment, the cross-sections will be summed two by two. In the semi-quantitative analysis, they will be summed five by five.

The "Brain Ratios" program will be employed for semi-quantitative image analysis. This program enables us to plot and locate elliptical, rectangular or irregular regions of interest (ROI) in the cerebral cortex, automatically, interactively or manually. It is also possible to apply ROI that have been stored from previous studies. The location and size of the different ROI can be modified, if necessary.

In the semi-automatic method, the operator selects a transaxial image that is reoriented in the orbital-meatal plane, centred on the central grey matter, and draws an ellipse to outline the external borders of the cerebral cortex. Then, within the ellipse, the program identifies 12 regions in the right cerebral hemisphere and 12 symmetric regions in the left cerebral hemisphere.

The following regions are specified: superior, middle and inferior frontal, anterior cingulated, inferior, middle and superior temporal, posterior/precuneous cingulated, thalamus, cerebellum, occipital cortex and left and right hemispheres, as well as the whole brain. The advantage of using this method is that it is not operator dependent. The program determines the number of pixels, the total counts and the activity normalized by surface (pixel of each ROI). It also determines the indices of the total counts and of the mean number of counts per pixel among each precise ROI and another one used as reference (left and right hemisphere, cerebellum, occipital cortex). The values for the regional brain perfusion in the post-acupuncture study will be obtained by calculating the number of total counts in each ROI, and subtracting from this value the decay-adjusted total number of counts for the same ROI in the pre-acupuncture study. The mean number of counts per pixel in each ROI will be normalized to the activity of the whole brain.

### Results measures

**Figure 1:**
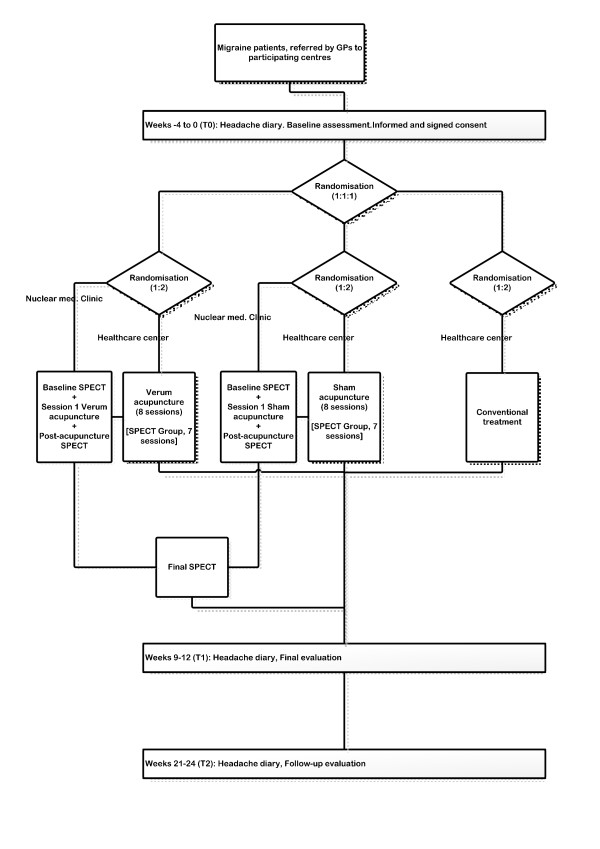
Trial work plan.

Each patient will fill in a headache diary during the 4 consecutive weeks immediately before the trial (baseline period), and during the 4 weeks following it (see figure 1). The diaries will be complemented with information on 4 additional weeks, from week 21 to week 24 after randomization (the follow-up period) Headache severity should be recorded in the diaries on a 3-point scale, by which 1 represents the presence of mild pain, 2 represents moderate pain and 3, severe pain (with incapacity to carry out everyday activities or necessity to remain in bed). The diary will also record the symptomatic and/or prophylactic medication taken, specifying the brand name and the number of doses. It should be clearly stated on the cover of the diary that the information contained within it is confidential. In addition, before starting the treatment, and also at 12 and 24 weeks after starting it, the patients will be requested to fill in a questionnaire with sociodemographic data and information concerning days off work because of headache; this will be compiled together with the Goldberg Anxiety-Depression Scale register and the results obtained from the Spanish version of the Headache Impact Test [36] and the SF-12 Health Survey (version 2). The purpose of this is to evaluate health-related aspects of the patient's quality of life [37]. The adverse effects of the techniques under study, and those of any rescue medication taken by the patient, will also be recorded. During the baseline evaluation, the patient will be asked to provide an anamnesis of the characteristics of the headache, in accordance with the recommendations of the International Headache Society, in order to classify the type of headache and to assess the term of the pain. Following this, an anamnesis and examination will be performed in accordance with the norms of traditional Chinese medicine.

#### Primary outcome measure

▪ The difference in the number of days with migraine (DWM) between the baseline period and the period spanning weeks 9–12 (DWM1).

#### Secondary outcome measures

▪ DWM between the baseline period and the period spanning weeks 21–24 (DWM2) after randomization.

▪ Change in the health-related quality of life between the baseline, final and follow-up periods, assessed by the SF-12 Health Survey.

▪ Days free from pain.

▪ Proportion of patients with at least 50% fewer headaches during the period spanning weeks 9–12 [33], in comparison with the baseline situation.

▪ Days off work, or of incapacity to perform daily activities.

▪ Change in the consumption of symptomatic/analgesic and prophylactic medication for migraine attacks, according to the patient's record of the name of the drug and the daily dose consumed, between the baseline period, the end of treatment and after six months.

▪ Change recorded on Goldberg's Depression/Anxiety Scale [38] between the baseline period, the end of treatment and after the follow-up period.

▪ Difference in the results of the Spanish version of the Headache Impact Test [39], the psychometric properties of which have been proven [36], between the baseline period, the end of treatment and after the follow-up period.

▪ SPECT: The following values will be calculated for the semi-quantitative analysis of the images:

• Percentage change of the mean value of counts per pixel for each region of interest, calculated as follows:

$$\%\text{ change}=100\times \frac{\text{post acupuncture}-\text{pre acupuncture}}{\text{pre acupuncture}}$$

• We shall also calculate an index of laterality (IL), as follows:

IL=(right−left)12×(right−left)

#### Evaluation of treatment credibility

Patients in Groups A and B will be asked, after the fourth treatment session, four items to be assessed on a continuous analogue visual scale from 0 to 10 (0 = totally disagree; 10 = totally agree): (1) Do you believe this treatment will reduce the pain you are suffering?; (2) Does the treatment seem to be a logical one?; (3) Would you recommend this treatment to a friend or relative with the same problem?; (4) Do you believe this treatment could be applicable to treat other problems? [40,41]

#### Sociodemographic variables

The following sociodemographic variables will be ascertained: date of birth, sex, marital status, level of education and race.

#### Variables related to the severity of the problem, occupational aspects and an estimation of direct tangible costs

Duration, in years, of the headache, its frequency, the duration of each attack and the degree of incapacity produced by the migraine.

Health utility will be measured in quality-adjusted life years gained by each patient, calculating the area under the curve of the SF-12 at 12 weeks after the start of treatment [42]. To estimate the costs of the different treatments, a map of activities will be created, and we will measure the costs that distinguish them (acupuncture, conventional treatment). The activities will be evaluated using an Activity Based Costing approach, according to which each activity consumes resources (inputs) – personal, materials and pharmaceutical – in order to produce results (outputs). These activities will be assessed one by one. The cost of each therapeutic treatment is defined as the sum of the costs of each of the activities that make it up. Thus we shall obtain a more accurate view of the cost variation produced by each treatment with respect to every other one, and will be able to study its impact on the following subprocess, as well as to make use of this information in future studies. The cost components of the activities addressed and the criteria applied to evaluate them in financial terms are as follows:

1. **Personal**. The consumption of human resources involved in carrying out the different activities. This evaluation includes the cost per hour of each category of professional staff. The cost is estimated taking into account the data provided by the Andalusia Public Health Service (SSPA), and includes both the explicit cost (wages and salaries paid) and the implicit cost (time travelling to and from the clinic, the environmental cost of this travelling, etc.).

2. **Occupational**. We will record the number of days off work, or incapacitated for the performance of routine tasks.

3. **Materials**. This includes the consumption of materials required for carrying out each activity. Each such material cost will be evaluated in accordance with the price recorded in the corresponding catalogue of the supplies department/service at the hospital or health district in question.

4. **Pharmaceuticals**. We shall consider the costs of the different pharmaceutical products (quantity and unit of measurement), evaluated in accordance with the normal price for sale to laboratories.

5. **Opportunity cost**. This will include the sum of the outputs of the potential activities that are not carried out as the result of applying the treatment, when an alternative might be used, and will include the potential financial cost of the resources employed.

On the basis of the ABC approach, the resources imputed to each patient can be applied, within a multidisciplinary table based on a modular matrix to act in the manner of a 'meter' for each patient, to the analysis model and thus we obtain the SF-12 curve showing the variation in the patient's quality of life and the potential cost savings for the public healthcare system, together with the increased years of life attained for each patient.

#### Characteristic diagnostic variables according to traditional Chinese medicine

The acupuncture physicians participating in the trial will be required to record the information needed to make a diagnosis according to traditional Chinese medicine, including the following factors: (1) the location of the headache; (2) the meridian or meridians most likely to be involved; (3) internal factors such as Blood, Qi, Yin, Yang, etc.; (4) external pathogens such as Wind, Phlegm, Cold, etc.; (5) the internal organs affected; (6) the state of internal body functions such as Stagnation, Excess, or Deficiency.

### Data compilation and analysis

#### Data compilation

A referral form has been drawn up, to be filled in by the GP requesting inclusion in the trial of patients who fulfil the selection criteria. A data base will be designed for the electronic storage of the variables recorded in the purpose-built (physical) data record book (DRB). This database (CRDD) will be kept at the analysis centre (in the Research Support Unit, Hospital Costa del Sol, Marbella. Malaga), which will be independent of the randomisation section. Each participating centre will have a duplicate of the structure of the CRDD, together with a codebook containing the definitions and operative characteristics of all the variables. This information will be compiled within an overall file containing the data for all the variables addressed in this trial, both in the user-controlled formats and in those determined by direct observation. These data will be recorded weekly in the CRDD and sent, in coded form, to the analysis centre for safekeeping. Neither the DRB nor the CRDD will identify the group to which the patient is allocated. This information will be controlled by the physician carrying out the treatments, and who will be responsible for liaising with the randomization centre. An external assessor will record all the information except that which is reserved for the medical staff, and will manage all aspects of the distribution of the patients; nevertheless, this assessor will be blind, at all times, to the treatment actually given to each patient. Every two weeks, the analysis centre will perform a quality control exercise on the data received.

#### Monitoring data and side effects

Records will be kept of all side effects that are reported, and possible adverse events caused by the experimental treatment or the medication provided.

#### Statistical analysis

Two types of population will be analysed: (1) population per intention to treat (ITT), with all the patients randomised; (2) population per protocol (PP), including only the patients presenting minor differences from the protocol. During analysis, the study groups will be anonymised in order to minimize biased reporting.

The baseline variables among the different groups will be compared to test the homogeneity produced by random allocation, in terms of the differences of means and proportions. The magnitude of the difference in the possible unbalancing produced by the random allocation among the groups will be evaluated using ratios of means and proportions (using that of the sham group as the reference level), and the final fit will be calculated by secondary analyses with multiple linear regression models. In the raw analysis (without fitting), significance tests will be used for the comparisons between *k *samples (parametric or not, depending on whether the distribution of the results variables is asymmetric or otherwise, and on the homogeneity of their variances), taking the control group (group C) as the reference level, and using comparison tests for the differences of the means in the main results variable (DWM1), both for the intergroup comparisons (for independent samples) and for the comparisons between the baseline and the final levels for each group (in the latter case, using tests for non-independent or paired samples).

For the main results variable (DWM1), linear regression models will be constructed, adjusted for the baseline level and analysing per ITT. The group variables will be included, taking the control group as a reference, together with the sociodemographic variables (age and sex) and the baseline variables of the severity of the complaint (pain intensity and frequency). We will adjust for possible confounders using criteria of statistical significance and of confusion. The detection of possible interactions with the treatment group variable will be evaluated by criteria of statistical significance for the corresponding interaction terms. The level of significance will be established at α < 0.05. The model will be reconstructed, removing all the observations with Cook's distances above the 90^th ^percentile of the distribution, in order to test the consistency of the results obtained.

It is our intention to perform an economic analysis from the standpoint of the Andalusia Public Health Service.

## Competing interests

The author(s) declare that they have no competing interests.

## Authors' contributions

JV conceived the study, designed the study protocol, sought funding and ethical approval and wrote the manuscript. All authors contributed to the research design, read, made critical revisions and approved the final manuscript.

## Pre-publication history

The pre-publication history for this paper can be accessed here:



## References

[B1] International Headache Society Classification Subcommittee (2004). The International Classification of Headache Disorders. Cephalalgia.

[B2] Monzon MJ, Lainez MJ (1998). Quality of life in migraine and chronic daily headache patients. Cephalalgia.

[B3] Fernandez-Concepcion O, Canuet-Delis L (2003). [Disability and quality of life in patients with migraine: determining factors]. Rev Neurol.

[B4] Martinez Eizaguirre JM, Calero MS, Garcia Fernandez ML, Tranche IS, Castillo OJ, Perez II (2006). [Attitudes of spanish primary care doctors to migraine]. Aten Primaria.

[B5] Badia X, Magaz S, Gutierrez L, Galvan J (2004). The burden of migraine in Spain: beyond direct costs. Pharmacoeconomics.

[B6] Stewart WF, Lipton RB, Celentano DD, Reed ML (1992). Prevalence of migraine headache in the United States. Relation to age, income, race, and other sociodemographic factors. JAMA.

[B7] Lipton RB, Bigal ME, Diamond M, Freitag F, Reed ML, Stewart WF (2007). Migraine prevalence, disease burden, and the need for preventive therapy. Neurology.

[B8] Hu XH, Markson LE, Lipton RB, Stewart WF, Berger ML (1999). Burden of migraine in the United States: disability and economic costs. Arch Intern Med.

[B9] Edmeads J, Findlay H, Tugwell P, Pryse-Phillips W, Nelson RF, Murray TJ (1993). Impact of migraine and tension-type headache on life-style, consulting behaviour, and medication use: a Canadian population survey. Can J Neurol Sci.

[B10] Garcia Monco JC, Alvarez de Arcaya A, Eguia del Rio P, Etxebarria Ibañez I, Gonzalez Hernandez N, Ruiz de Velasco Artaza I (2002). Revision sobre el tratamiento farmacológico de la migraña y estudio cualitativo.

[B11] Garcia-Escriva A, Asensio-Asensio M, Lopez-Hernandez N, Gonzalez-Aznar OJ, Oliver-Navarrete C, Alvarez-Sauco M, Pampliega-Perez A (2004). [Health care activity in a headache-specific clinic]. Rev Neurol.

[B12] Beckett BE, Herndon KC, Dipiro JT, Talbert RL, Yee GC, Matzke GR, Wells BG, Posey LM (2002). Headache disorders. Pharmacotherapy: A Pathophysiologic Approach.

[B13] Linde K, Streng A, Jurgens S, Hoppe A, Brinkhaus B, Witt C, Wagenpfeil S, Pfaffenrath V, Hammes MG, Weidenhammer W, Willich SN, Melchart D (2005). Acupuncture for patients with migraine: a randomized controlled trial. JAMA.

[B14] Vickers AJ, Rees RW, Zollman CE, McCa rney R, Smith CM, Ellis N, Fisher P, Van HR (2004). Acupuncture for chronic headache in primary care: large, pragmatic, randomised trial. BMJ.

[B15] Melchart D, Linde K, Fischer P, Berm an B, White A, Vickers A, Allais G (2001). Acupuncture for idiopathic headache. Cochrane Database Syst Rev.

[B16] Diener HC, Kronfeld K, Boewing G, Lungenhausen M, Maier C, Molsberger A, Tegenthoff M, Trampisch HJ, Zenz M, Meinert R (2006). Efficacy of acupuncture for the prophylaxis of migraine: a multicentre randomised controlled clinical trial. Lancet Neurol.

[B17] Endres HG, Diener HC, Molsberger A (2007). Role of acupuncture in the treatment of migraine. Expert Rev Neurother.

[B18] Tatsch K, Asenbaum S, Bartenstein P, Catafau A, Halldin C, Pilowsky LS, Pupi A (2002). European Association of Nuclear Medicine procedure guidelines for brain perfusion SPET using (99m)Tc-labelled radiopharmaceuticals. Eur J Nucl Med Mol Imaging.

[B19] Juni JE, Waxman AD, Devous MD, Tikofsky RS, Ichise M, Van Heertum RL, Holman BL, Carretta RF, Chen CC (1998). Procedure guideline for brain perfusion SPECT using technetium-99m radiopharmaceuticals. Society of Nuclear Medicine. J Nucl Med.

[B20] Blake P, Johnson B, VanMeter JW (2003). Positron Emission Tomography (PET) and Single Photon Emission Computed Tomography (SPECT): Clinical Applications. J Neuroophthalmol.

[B21] Lauritzen M, Olesen J (1984). Regional cerebral blood flow during migraine attacks by Xenon-133 inhalation and emission tomography. Brain.

[B22] Podreka I, Suess E, Goldenberg G, Steiner M, Brucke T, Muller C, LANG W, Neirinckx RD, Deecke L (1987). Initial experience with technetium-99m HM-PAO brain SPECT. J Nucl Med.

[B23] Levine SR, Welch KM, Ewing JR, Robertson WM (1987). Asymmetric cerebral blood flow patterns in migraine. Cephalalgia.

[B24] Friberg L, Olesen J, Iversen H, Nicolic I, Sperling B, Lassen NA, Olsen TS, Tfelt-Hansen P (1994). Interictal "patchy" regional cerebral blood flow patterns in migraine patients. A single photon emission computerized tomographic study. Eur J Neurol.

[B25] Facco E, Munari M, Baratto F, Behr AU, Dal PA, Cesaro S, Giacomini M, Giron G (1996). Regional cerebral blood flow (rCBF) in migraine during the interictal period: different rCBF patterns in patients with and without aura. Cephalalgia.

[B26] De Benedittis G, Ferrari Da PC, Granata G, Lorenzetti A (1999). CBF changes during headache-free periods and spontaneous/induced attacks in migraine with and without aura: a TCD and SPECT comparison study. J Neurosurg Sci.

[B27] Schlake HP, Bottger IG, Grotemeyer KH, Husstedt IW, Vollet B, Schober O, Brune GG (1990). Single photon emission computed tomography with technetium-99m hexamethyl propylenamino oxime in the pain-free interval of migraine and cluster headache. Eur Neurol.

[B28] Lee JD, Chon JS, Jeong HK, Kim HJ, Yun M, Kim DY, Kim DI, Park CI, Yoo HS (2003). The cerebrovascular response to traditional acupuncture after stroke. Neuroradiology.

[B29] Wang F, Jia SW (1996). [Effect of acupuncture on regional cerebral blood flow and cerebral functional activity evaluated with single-photon emission computed tomography]. Zhongguo Zhong Xi Yi Jie He Za Zhi.

[B30] Newberg AB, Lariccia PJ, Lee BY, Farrar JT, Lee L, Alavi A (2005). Cerebral blood flow effects of pain and acupuncture: a preliminary single-photon emission computed tomography imaging study. J Neuroimaging.

[B31] Ha-Kawa SK, Yoshida T, Yague T, Tani M, Suzuki T, Sawada S (2006). Acupuncture-induced cerebral blood flow responses in dystonia. Ann Nucl Med.

[B32] Campbell A (2006). Point specificity of acupuncture in the light of recent clinical and imaging studies. Acupunct Med.

[B33] Tfelt-Hansen P, Block G, Dahlof C, Diener HC, Ferrari MD, Goadsby PJ, Guidetti V, Jones B, Lipton RB, Massiou H, Meinert C, Sandrini G, Steiner T, Winter PB (2000). Guidelines for controlled trials of drugs in migraine. Cephalalgia.

[B34] Grupo de Estudio de Cefaleas de la Sociedad Española de Neurologia (2004). Actitud diagnóstica y terapéutica en la cefalea: Recomendaciones.

[B35] Coeytaux RR, Chen W, Lindemuth CE, Tan Y, Reilly AC (2006). Variability in the diagnosis and point selection for persons with frequent headache by traditional Chinese medicine acupuncturists. J Altern Complement Med.

[B36] Martin M, Blaisdell B, Kwong JW, Bjorner JB (2004). The Short-Form Headache Impact Test (HIT-6) was psychometrically equivalent in nine languages. J Clin Epidemiol.

[B37] Ware E, Leplege A, Sullivan M, Gandek B, Aaronson N, Alonso J, Apolone G, rner J, Braiser J, Bullinger M, Kaasa S (1997). Testing the SF-12 summary health measures in nine countries: Results from the IQOLA project. Quality Life Res.

[B38] Monton C, Perez Echeverria MJ, Campos R, Garcia CJ, Lobo A (1993). [Anxiety scales and Goldberg's depression: an efficient interview guide for the detection of psychologic distress]. Aten Primaria.

[B39] Gandek B, Alacoque J, Uzun V, ndrew-Hobbs M, Davis K (2003). Translating the Short-Form Headache Impact Test (HIT-6) in 27 countries: methodological and conceptual issues. Qual Life Res.

[B40] Vincent C, Lewith G (1995). Placebo controls for acupuncture studies. J R Soc Med.

[B41] Borkovec TD, Nau SD (1972). Credibility of analogue therapy rationales. J Beh Ther Exp Pschiat.

[B42] Richardson G, Manca A (2004). Calculation of quality adjusted life years in the published literature: a review of methodology and transparency. Health Econ.

